# Secondhand smoke exposure and behavioral problems in Japanese schoolchildren

**DOI:** 10.3389/fpubh.2025.1595509

**Published:** 2025-07-03

**Authors:** Toshiki Hata, Haruhi Ishida, Hiroko Inoue, Ayako Hashimoto, Yukina Morimoto, Ikuko Nagaya, Kozue Nakamura, Toshiko Kuwano

**Affiliations:** ^1^Graduate School of Integrated Pharmaceutical and Nutritional Sciences, University of Shizuoka, Shizuoka, Japan; ^2^Department of Nutrition and Health Sciences, Faculty of Food and Nutritional Sciences, Toyo University, Asaka, Japan; ^3^Department of Food and Nutrition, Faculty of Home Economics, Kyoto Women’s University, Kyoto, Japan; ^4^Department of Food and Nutrition, Gifu City Women’s College, Gifu, Japan; ^5^Gifu City Health Center, Gifu, Japan

**Keywords:** secondhand smoke, behavioral problems, SDQ, schoolchildren, Japan, urinary cotinine, child mental health

## Abstract

**Introduction:**

Evidence on the association between secondhand tobacco smoke exposure and behavioral problems in schoolchildren remain limited. This study examined the association between objectively measured secondhand smoke exposure and behavioral problems among Japanese schoolchildren.

**Methods:**

This study included 892 schoolchildren (444 boys, 448 girls) in Yamagata City, Gifu, Japan. Secondhand smoke exposure was assessed using urinary creatinine-corrected cotinine concentration (UC), with levels <5.0 ng/mgCr classified as low and ≥5.0 ng/mgCr as high. Behavioral problems were evaluated using the Strengths and Difficulties Questionnaire (SDQ), completed by guardians. Multivariate-adjusted odds ratios (ORs) and 95% confidence intervals (CIs) were calculated by grade level.

**Results:**

Among participants, 10.1% (*n* = 41) in the early grades and 7.4% (*n* = 36) in the upper grades had high UC. In the upper grades, the OR for behavioral problems was significantly higher in those with high UC (OR: 2.39, 95% CI: 1.05–5.45). Additionally, Hyperactivity, Conduct problems, and Prosocial behavior were significantly worse in students with high UC. However, in the early grades, no significant association was observed between high UC and behavioral problems (OR: 1.04, 95% CI: 0.45–2.40), except for emotional symptoms, which were significantly worse in children with high UC.

**Discussion:**

Secondhand smoke exposure may increase the risk of behavioral problems in Japanese schoolchildren, particularly in the upper grades. Reducing exposure, especially at home and in the community, should be prioritized to prevent behavioral and psychological problems.

## 1 Introduction

Childhood developmental and behavioral problems can have lifelong and intergenerational effects (1, 2). Thus, addressing these problems early in life is essential for improving health during adulthood, including old age (3–5). Children’s mental health problems, often manifested as behavioral issues, are increasing globally and impose a significant burden on society (6). Epidemiological studies indicate that up to 20% of children experience debilitating mental disorders, and nearly 50% of adult mental disorders have their onset in adolescence (7). Moreover, children’s mental health problems can contribute to lower educational attainment, including early school leaving and school refusal, thereby diminishing future socioeconomic opportunities (8). Consequently, preventive interventions targeting childhood mental health disorders are essential.

Several studies have identified secondhand smoke exposure as an environmental factor associated with behavioral problems in children (9–20). Notably, childhood exposure to secondhand smoke has been linked to conditions such as attention deficit hyperactivity disorder (ADHD), conduct disorder, and disruptive behavior (11, 13–17, 19, 20). Secondhand smoke has also been reported to adversely affect the development of cranial nerve function, underscoring the need to prevent exposure (21–23). A widely used tool to assess children’s behavioral problems is the Strengths and Difficulties Questionnaire (SDQ) (24), a brief and validated screening instrument commonly applied in community, clinical, and research settings to evaluate both externalizing and internalizing symptoms (25–27).

Although previous research has explored the association between secondhand smoke exposure and behavioral problems assessed using the SDQ, studies in Japan remain limited, particularly those focusing on school-aged children rather than preschoolers (20). Therefore, this study aimed to examine the association between secondhand smoke exposure and behavioral problems, assessed using the SDQ, among schoolchildren living in a medium-altitude mountainous region of Japan.

## 2 Materials and methods

### 2.1 Study participants

**Figure 1:**
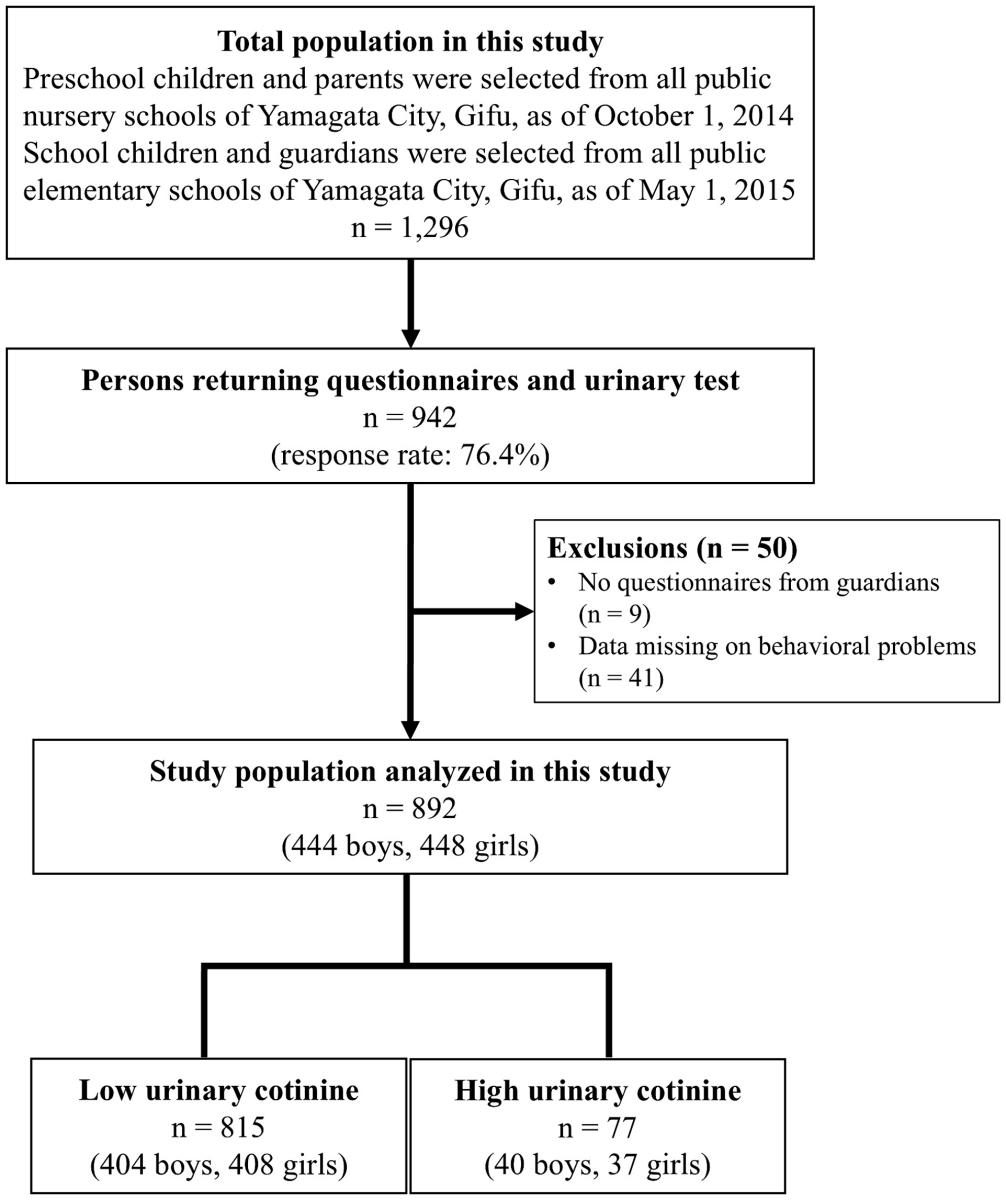
Flow diagram of the study participants.

This study was conducted in all public nursery and elementary schools in Yamagata City, Gifu, Japan. Participants included senior preschool children (aged 5–6 years), elementary school students from grades 2 to 6 (aged 7–12 years), and their guardians. In October 2014 (senior preschool children) and May–June 2015 (elementary school students), 1,296 self-administered questionnaires were distributed to children and their guardians, requesting first-void morning urine samples. A total of 942 questionnaires were returned, yielding a response rate of 76.4%. Finally, 892 questionnaires with urine samples (444 boys and 448 girls) were included, as these had complete guardian questionnaires and behavioral problem assessments (Figure 1).

The study protocol was developed in accordance with the ethical principles outlined in the Declaration of Helsinki and was approved by the Ethics Committee of the University of Shizuoka (No. 26–3). Informed consent was obtained from all participants before enrollment. The study is registered in the UMIN Clinical Trials Registry (UMIN000033146).

### 2.2 Measurements

#### 2.2.1 Secondhand smoke status

Children’s secondhand smoke exposure was defined based on urinary total cotinine concentrations (28). First-void morning urine samples were collected at home upon waking and brought to nursery and elementary schools, where they were immediately transported to the laboratory. Upon arrival, urine samples were aliquoted and stored at −80°C until analysis. To account for urine dilution variability, the creatinine-corrected cotinine concentration was calculated as the ratio of cotinine to creatinine concentrations (ng/mg Cr) (29). Urinary cotinine levels (ng/dL) were measured using the cotinine enzyme-linked immunosorbent assay kit for secondhand smoke (Cosmic Corporation Co., Ltd., Tokyo, Japan). Urinary creatinine concentrations (mg/dL) were measured via an enzymatic method by SRL Diagnostics Inc. (Tokyo, Japan). In this study, the urinary creatinine-corrected cotinine concentration (UC) cut-off was set at 5.0 ng/mgCr, with UC levels classified as low (<5.0 ng/mgCr) and high (≥5.0 ng/mgCr) (30–32).

#### 2.2.2 Behavioral problems

The SDQ is a validated instrument used to assess both positive and negative behavioral attributes in children. It consists of 25 items categorized into 5 subscales: Peer problems, Hyperactivity, Conduct problems, Emotional symptoms, and Prosocial behavior (24). The SDQ is widely used as a screening tool for behavioral problems in pediatric populations. Each subscale is scored on a scale of 0 to 10, with higher scores indicating greater severity. The total difficulty score (TDS) was calculated as the sum of the Peer problems, Hyperactivity, Conduct problems, and Emotional symptoms subscale scores, with a total possible range of 0 to 40. Previous studies have demonstrated that SDQ-TDS varies by age among Japanese children, and behavioral problems are defined as the highest 20th percentile of the normative sample (20, 24, 33, 34). In this study, a score ≥15 was considered indicative of behavioral problems for the early grades, while a score ≥14 was used for the upper grades.

#### 2.2.3 Covariates

The covariates in this study included age, sex, Rohrer index (<115, 115–144.9, or ≥145), medical history of asthma, medical history of allergic rhinitis, family composition (nuclear family: present or absent), smoking by cohabiting family members, educational attainment of guardians (≥Junior college/college: present or absent), and social nicotine dependence of guardians. The Rohrer index was calculated using the formula: body weight (kg) divided by the cube of height (cm^3^) and multiplied by 10^7^. The index was categorized into thinness (<115) and obesity (≥145). The social nicotine dependence of guardians was assessed using the Kano test for social nicotine dependence (KTSND), a validated 10-item questionnaire with a total possible score ranging from 0 to 30 (35). Participants scoring ≥10 were classified as highly socially nicotine-dependent, while those scoring ≤9 were classified as having low social nicotine dependence (36).

### 2.3 Statistical analyses

Data were analyzed using Stata 18.0 (StataCorp, TX, USA). A significance level of *α* = 0.05 was used to determine statistical significance. To examine differences in continuous variables associated with secondhand smoke exposure, the Mann–Whitney U test was used. For categorical variables, the chi-square test was conducted. The relationship between secondhand smoke exposure and behavioral problems was assessed using a generalized linear mixed model (GLMM), considering the multilevel data structure, where individuals (at Level 1) were nested within 3 living areas (at Level 2). The GLMM was applied using fixed slopes and random intercept models, calculating the estimated parameters (*β*) and 95% confidence intervals (CIs) for the total SDQ score, the 5 SDQ subscales, and SDQ-TDS by grade. Similarly, multilevel logistic regression analyses were conducted to calculate the adjusted odds ratios (ORs) and 95%CIs for behavioral problems by grade. In both models, secondhand smoke exposure was included as a fixed factor, while the living area was treated as a random factor. The adjusted models included all covariates. The goodness of fit of the multilevel analysis was confirmed by the intraclass correlation coefficient and the likelihood ratio test. Multicollinearity was checked using the tolerance and variance inflation factors.

For secondary analyses assessing the influence of sex, stratified analyses were conducted using the same statistical approach, separately for boys and girls.

Participants with missing data on secondhand smoke exposure or SDQ scores were excluded from the analysis. Missing covariates were assigned to a “missing” category and included in the analysis to minimize selection bias.

## 3 Results

Table 1 presents the characteristics of the study population based on urinary cotinine levels, stratified by grade. The mean urinary cotinine concentration was 2.3 ± 6.0 ng/mgCr in the early grades and 1.5 ± 5.2 ng/mgCr in the upper grades. Among the early grades, 41 (10.1%) were classified as having high UC, while among upper-grade students, 36 (7.4%) were classified as having high UC. Significant differences were observed between high and low UC groups in the early grades with respect to age, Rohrer index, smoking by cohabiting family members, guardians’ educational attainment, and KTSND scores. Similarly, in the upper grades, significant differences were found in Rohrer index, medical history of allergic rhinitis, smoking by cohabiting family members, and KTSND scores.

**Table 1:** Baseline characteristics of study participants, according to urinary cotinine category by school grade.

Variables	Early grades (*N* = 405)							Upper grades (*N* = 487)						
	All		Low UC		High UC		p*	All		Low UC		High UC		p*
			(*n* = 364, 89.9%)		(*n* = 41, 10.1%)					(*n* = 451, 92.6%)		(*n* = 36, 7.4%)		
Urinary Cotinine (ng/mgCr), mean (SD)	2.3	(6.0)	0.9	(1.1)	14.6	(13.2)	<0.001	1.5	(5.2)	0.5	(0.9)	14.2	(13.7)	<0.001
Age (years), mean (SD)	7.1	(1.2)	7.1	(1.2)	6.5	(1.2)	0.005	10.2	(0.9)	10.2	(0.9)	10.4	(1.0)	0.209
Sex (boys), *n* (%)	210	(51.9)	189	(51.9)	21	(51.2)	0.932	234	(48.0)	215	(47.7)	19	(52.8)	0.563
Rohrer index, mean (SD)	129.6	(15.3)	128.9	(15.1)	137.1	(15.4)	0.008	123.3	(19.4)	122.5	(18.6)	135.4	(26.6)	0.005
<115 (thinness), *n* (%)	60	(14.8)	59	(16.2)	1	(2.4)	0.016	145	(29.8)	140	(31.0)	5	(13.9)	0.022
≥145 (obesity), *n* (%)	48	(11.9)	38	(10.4)	10	(24.4)	0.001	45	(9.2)	36	(8.0)	9	(25.0)	<0.001
Medical history of asthma (presence), *n* (%)	108	(26.7)	97	(26.7)	11	(26.8)	0.978	192	(39.4)	178	(39.5)	14	(38.9)	0.980
Medical history of allergic rhinitis (presence), *n* (%)	48	(11.9)	43	(11.8)	5	(12.2)	0.991	65	(13.3)	55	(12.2)	10	(27.8)	0.030
Family composition (nuclear family), *n* (%)	268	(66.2)	246	(67.6)	22	(53.7)	0.074	313	(64.3)	293	(65.0)	20	(55.6)	0.257
Smoking by family members living together (presence), *n* (%)	189	(46.7)	154	(42.3)	35	(85.4)	<0.001	208	(42.7)	181	(40.1)	27	(75.0)	<0.001
Education level of guardians (≥Junior college/college), *n* (%)	220	(54.3)	206	(56.6)	14	(34.2)	0.003	224	(46.0)	210	(46.6)	14	(38.9)	0.630
KTSND of guardians (points), mean (SD)	10.7	(5.1)	10.4	(5.0)	13.1	(5.6)	0.004	10.9	(5.4)	10.7	(5.2)	14.6	(5.9)	<0.001
≥10, *n* (%)	239	(59.0)	211	(58.0)	28	(68.3)	0.317	287	(58.9)	260	(57.7)	27	(75.0)	0.053

UC, urinary cotinine; SDQ, strengths and difficulties questionnaire; TDS, total difficult score; KTSND, kano test for social nicotine dependence. *Mann–Whitney U-test or chi-square test comparing low UC vs. high UC groups.

Table 2 presents the GLMM estimates for SDQ subscales and SDQ-TDS, stratified by grade. In the early grades, students with high UC exhibited significantly worse scores on Emotional Symptoms (0.36 points; 95% CI, 0.09–0.63). In the upper grades, high UC was significantly associated with higher SDQ-TDS (0.23 points; 95% CI, 0.07–0.40), Hyperactivity (0.30 points; 95% CI, 0.12–0.48), Conduct problems (0.30 points; 95% CI, 0.10–0.49), and lower Prosocial behavior (−0.14 points; 95% CI, −0.27 to −0.02).

**Table 2:** Generalized linear mixed model estimated for SDQ as urinary cotinine by school grade.

Categories	Estimated parameters β (95% CI)					
	TDS	Peer problems	Hyperactivity	Conduct problems	Emotional symptoms	Prosocial behavior
Early grades (*N* = 405)						
Low UC	10.17 points	1.87 points	3.85 points	2.56 points	1.81 points	6.14 points
High UC	0.08 (−0.07, 0.24)	0.16 (−0.08, 0.40)	−0.06 (−0.25, 0.14)	−0.03 (−0.25, 0.19)	0.36 (0.09, 0.63)	0.09 (−0.02, 0.63)
	p* = 0.286	p* = 0.191	p* = 0.581	p* = 0.755	p* = 0.008	p* = 0.128
Upper grades (*N* = 487)						
Low UC	8.92 points	2.00 points	3.09 points	2.02 points	1.88 points	6.47 points
High UC	0.23 (0.07, 0.40)	0.21 (−0.02, 0.43)	0.30 (0.10, 0.48)	0.30 (0.10, 0.49)	0.12 (−0.16, 0.41)	−0.14 (−0.27, −0.02)
	p* = 0.004	p* = 0.069	p* = 0.001	p* = 0.003	p* = 0.397	p* = 0.028

UC, urinary cotinine; SDQ, strengths and difficulties questionnaire; TDS, total difficulties score; CI, confidence interval. Adjusted for age, sex, thinness, obesity, asthma, allergic rhinitis, family composition, smoking by family members in the household, education level of guardians, and KTSND of guardians.

Table 3 presents the associations between urinary cotinine levels and behavioral problems, stratified by grade. Among the early grades, high UC (OR, 1.04; 95% CI, 0.45–2.40) was not significantly associated with behavioral problems. However, among the upper grades, high UC (OR, 2.39; 95% CI, 1.05–5.45) was significantly associated with behavioral problems.

**Table 3:** Multilevel logistic regressions of associations of urinary cotinine with SDQ-TDS by school grade.

Categories	Number of some or high need per participants	Crude			Multi-variables adjusted model*		
		OR	(95% CI)	*p*	OR	(95% CI)	*p*
Early grades (*N* = 405)							
Low UC	71/364	1.00	(Ref.)		1.00	(Ref.)	
High UC	11/41	1.51	(0.72, 3.16)	0.271	1.04	(0.45, 2.40)	0.934
Upper grades (*N* = 487)							
Low UC	78/451	1.00	(Ref.)		1.00	(Ref.)	
High UC	14/36	3.04	(1.49, 6.21)	0.002	2.39	(1.05, 5.45)	0.038

UC, urinary cotinine; SDQ, strengths and difficulties questionnaire; TDS, total difficulties score; CI, confidence interval, OR: odds ratio. *Adjusted for age, sex, thinness, obesity, Rohrer index, asthma, allergic rhinitis, family composition, smoking by family members in the household, education level of guardians, and KTSND of guardians.

The results of the SDQ subscales and SDQ-TDS stratified analyses are presented in Supplementary Tables 1, 2. For boys, the results were consistent with those observed in the primary analysis. Similar results were obtained for girls, although some of the significance disappeared.

Supplementary Table 3 presents the comparison results for the association between UC and behavioral problems stratified by sex and school grade. The results were similar to those observed in the primary analysis, although significance disappeared for both sexes.

## 4 Discussion

This study examined the association between secondhand smoke exposure and behavioral problems in a cross-sectional analysis of children living in a medium-altitude mountainous region of Japan. The findings indicated that urinary cotinine levels were not significantly associated with SDQ scores in the early grades. However, in the upper grades, higher urinary cotinine levels were associated with increased SDQ scores and a greater risk of behavioral problems. These results suggest that secondhand smoke exposure is linked to behavioral problems in children, particularly in upper grades.

Among the upper grades, higher urinary cotinine levels were significantly associated with increased SDQ-TDS, Hyperactivity, and Conduct Problems, as well as lower prosocial behavior. In contrast, in the early grades, higher urinary cotinine levels were associated with only emotional symptoms and were not linked to overall behavioral problems. Exposure to secondhand tobacco smoke in experimental animals resulted in cellular and neuronal projection damage (22), upregulation of nicotinic cholinergic receptors (24), and alterations in serotonin synaptic proteins within the brain (23). These changes may exert toxic effects on the developing brain. Previous studies that objectively assessed secondhand smoke exposure and behavioral problems in children reported that higher salivary cotinine levels were positively associated with behavioral problems (15, 19). Furthermore, studies measuring serum cotinine found a positive association between elevated cotinine levels and increased risk of behavioral problems (16–18). Additionally, higher urinary cotinine levels were linked to increased SDQ-TDS (20) and the occurrence of ADHD and ASD in early-grade students (37). Although the findings for upper grades in this study align with previous research, the results for the early grades differ. A previous study observed that children exposed to secondhand smoke at home were at a higher risk of developing neurobehavioral disorders, with boys and older children being more affected (38). Similar results were obtained in the present study, with the lower effect of secondhand smoke in the younger ages possibly attributable to genetic effects of the prenatal environment. Prior studies on infants reported no significant association between salivary cotinine concentrations and SDQ-TDS (39), suggesting the need for further research on the relationship between passive smoking and behavioral problems in younger children.

The mean (standard deviation) urinary cotinine concentration in this study was 2.3 (6.0) ng/mgCr in the early grades and 1.5 (6.0) ng/mgCr in the upper grades. Additionally, 10.1% of the early grades and 7.4% of the upper grades were classified as having high exposure to secondhand smoke, defined as urinary cotinine levels ≥5.0 ng/mgCr. The smoking rate among cohabiting family members of the study participants was 44.5%, which is relatively high given that the national smoking rate in Japan was 21.6% (33.7% for men and 10.7% for women), and the overall smoking rate in Gifu Prefecture was 20.5% (32.4% for men and 9.7% for women) according to the 2013 Comprehensive Survey of Living Conditions (40). Therefore, the smoking rate among the guardians of the children in this study was substantially higher than the national and regional averages. The mean SDQ-TDS was 10.3 (5.4) points in the early grades and 9.1 (5.4) points in the upper grades. A previous study reported that among Japanese children, the mean SDQ-TDS for ages 7–9 years was 8.4 (5.1) points, while for ages 10–12 years, it was 7.1 (5.1) points (34). Thus, the SDQ-TDS in this study were approximately 2 points higher than the reported national averages. These findings suggest that the study participants may have been exposed to higher levels of secondhand smoke compared with the general population, which may have contributed to the relatively elevated SDQ-TDS scores and increased prevalence of behavioral problems.

This study has several limitations. First, its cross-sectional design prevents the establishment of causal relationships between secondhand smoke exposure and behavioral problems. However, previous research has shown that young children exposed to secondhand smoke have an increased risk of developing behavioral problems later in life (11, 12). Similar patterns are likely in this study population. Second, the self-administered questionnaire may have introduced recall bias since children’s behavioral status was reported by guardians. Nevertheless, the parental version of the SDQ has been validated for screening conduct disorder and ADHD in preschool-aged children and reliably predicts clinical outcomes 2 years later (41). Third, the presence of unmeasured confounding variables such as comorbidities and neurological diagnoses cannot be ruled out, which may have influenced the results and potentially led to an overestimation of associations. Fourth, this study was conducted in a medium-altitude mountainous region where smoking rates may differ from other parts of Japan. Given regional variations in smoking prevalence (40), further research should include metropolitan areas with lower smoking rates to assess whether similar associations persist across different environments. Finally, in recent years, the effect of tobacco smoke pollutants that remain in the indoor environment, the so-called “third-hand smoke,” on the health status of children have been reported (42, 43). However, residual substances were not investigated in this study, and thus the effects of third-hand smoke could not be determined. A previous study showed that cotinine levels in children’s hands, one of the indicators of third-hand smoke, are positively correlated with urinary cotinine levels (44). For this reason, the effect of third-hand smoke on the participants in the present study may be comparable to those of secondhand smoke.

Despite these limitations, to the best of our knowledge, this is the first study among Japanese schoolchildren to demonstrate that reducing secondhand smoke exposure may lower the risk of behavioral problems.

In conclusion, secondhand smoke exposure increases the risk of behavioral problems in Japanese schoolchildren. Preventing exposure is essential and can be achieved through stronger public health initiatives promoting smoke-free environments at home and in broader community settings.

## Data Availability

The raw data supporting the conclusions of this article will be made available by the authors, without undue reservation.
