# Supplementary material for: Secondhand smoke exposure and behavioral problems in Japanese schoolchildren
Source: Front Public Health. 2025 Jul 3;13:1595509. doi: 10.3389/fpubh.2025.1595509 (PMC12267285; doi:10.3389/fpubh.2025.1595509)
Supplement: Supplementary file 1 [file Table_1.docx]

Supplementary Material

# 1 Supplementary Figures and Tables

## Supplementary Tables

| **Supplementary Table 1**. Generalized linear mixed model estimates for Strengths and Difficulties Questionnaire (SDQ) as a function of urinary cotinine levels among boys, stratified by school grade | | | | | | |
| --- | --- | --- | --- | --- | --- | --- |
|  | Estimated parameters β (95% CI) | | | | | |
|  | TDS | Peer problems | Hyperactivity | Conduct problems | Emotional symptoms | Prosocial behavior |
| **Early grades (*n* = 210)** | | | | | | |
| Low UC | 10.59 points | 1.85 points | 4.27 points | 2.67 points | 1.84 points | 5.70 points |
| High UC | 0.15 (-0.05, 0.35) | 0.10 (-0.21, 0.41) | 0.02 (-0.23, 0.27) | 0.07 (-0.21, 0.36) | 0.51 (0.17, 0.86) | 0.02 (-0.16, 0.20) |
|  | *P** = 0.148 | *P** = 0.533 | *P** = 0.870 | *P** = 0.624 | *P** = 0.003 | *P** = 0.856 |
| **Upper grades (*n* = 234)** | | | | | | |
| Low UC | 9.42 points | 2.08 points | 3.61 points | 2.13 points | 1.69 points | 5.93 points |
| High UC | 0.27 (0.04, 0.50) | 0.15 (-0.16, 0.47) | 0.36 (0.12, 0.60) | 0.43 (0.16, 0.70) | 0.14 (-0.28, 0.56) | -0.21 (-0.42, -0.00) |
|  | *P** = 0.019 | *P** = 0.344 | *P** = 0.004 | *P** = 0.002 | *P** = 0.509 | *P** = 0.046 |
| UC: urinary cotinine; SDQ: strengths and difficulties questionnaire; TDS: total difficulties score; CI: confidence interval  *: Adjusted for age, thinness, obesity, asthma, allergic rhinitis, family conposition, smoking by family members in the household,  education level of guardians, and KTSND of guardians. | | | | | | |

| **Supplementary Table 2**. Generalized linear mixed model estimates for Strengths and Difficulties Questionnaire (SDQ) as a function of urinary cotinine levels among girls, stratified by school grade | | | | | | |
| --- | --- | --- | --- | --- | --- | --- |
|  | Estimated parameters β (95% CI) | | | | | |
|  | TDS | Peer problems | Hyperactivity | Conduct problems | Emotional symptoms | Prosocial behavior |
| **Early grades (*n* = 195)** | | | | | | |
| Low UC | 9.44 points | 1.81 points | 3.36 points | 2.56 points | 1.91 points | 6.56 points |
| High UC | 0.03 (-0.20, 0.26) | 0.21 (-0.16, 0.58) | -0.15 (-0.49, 0.19) | -0.12 (-0.44, 0.21) | 0.16 (-0.26, 0.58) | 0.13 (-0.02, 0.28) |
|  | *P** = 0.793 | *P** = 0.271 | *P** = 0.383 | *P** = 0.486 | *P** = 0.462 | *P** = 0.082 |
| **Upper grades (*n* = 253)** | | | | | | |
| Low UC | 8.29 points | 1.87 points | 2.54 points | 1.81 points | 2.06 points | 6.89 points |
| High UC | 0.26 (0.03, 0.48) | 0.33 (0.02, 0.63) | 0.32 (0.04, 0.60) | 0.11 (-0.25, 0.47) | 0.17 (-0.18, 0.53) | -0.09 (-0.24, 0.07) |
|  | *P** = 0.028 | *P** = 0.035 | *P** = 0.028 | *P** = 0.564 | *P** = 0.343 | *P** = 0.275 |
| UC: urinary cotinine; SDQ: strengths and difficulties questionnaire; TDS: total difficulties score; CI: confidence interval  *: Adjusted for age, thinness, obesity, asthma, allergic rhinitis, family conposition, smoking by family members in the household,  education level of guardians, and KTSND of guardians. | | | | | | |

| **Supplementary Table 3**. Multilevel logistic regression models examining associations between urinary cotinine levels and Strengths and Difficulties Questionnaire-total difficulty score (SDQ-TDS), stratified by sex and school grade | | | | | | | | | | | | | | | |
| --- | --- | --- | --- | --- | --- | --- | --- | --- | --- | --- | --- | --- | --- | --- | --- |
|  | **Early grades** | | | | | | |  | **Upper grades** | | | | | | |
|  | Boys (*n* = 210) | | |  | Girls (*n* = 195) | | |  | Boys (*n* = 234) | | |  | Girls (*n* = 253) | | |
|  | OR | (95% CI) | *P** |  | OR | (95% CI) | *P** |  | OR | (95% CI) | *P** |  | OR | (95% CI) | *P** |
| Low UC | 1.00 | (Ref.) |  |  | 1.00 | (Ref.) |  |  | 1.00 | (Ref.) |  |  | 1.00 | (Ref.) |  |
| High UC | 1.87 | (0.60, 5.83) | 0.280 |  | 1.59 | (0.54, 5.02) | 0.490 |  | 2.56 | (0.72, 9.03) | 0.145 |  | 3.02 | (0.84, 10.84) | 0.090 |
| UC: urinary cotinine; SDQ: strengths and difficulties questionnaire; TDS: total difficulties score; CI: confidence interval, OR: odds ratio  *: Adjusted for age, thinness, obesity, Rohrer index, asthma, allergic rhinitis, family conposition,  smoking by family members in the household, education level of guardians, and KTSND of guardians. | | | | | | | | | | | | | | | |
